# Naturally occurring benzophenones and xanthones from *Garcinia smeathmannii* (Planch. & Triana) Oliv. displayed anti-inflammatory effects by modulating the activities of inflammatory mediators in LPS-stimulated RAW 264.7 macrophages

**DOI:** 10.3389/fphar.2024.1370073

**Published:** 2024-06-03

**Authors:** Mohammed Jeelani, Hugues Fouotsa, Osama A. Mohammed, Jaber Alfaifi, Salmon Adebayo, Mohammad Muzammil Ahmed, Amar Ibrahim Omer Yahia, Hanan Eissa, Emad Bahashwan, Nahid Ahmed Mohammed, Yousef Ayesh Alotaibi, Ashwaq Yahya Asiri, Assad Rezigallah, Muffarah Hamid Alharthi, Jean Paul Dzoyem, Adamu Imam Isa

**Affiliations:** ^1^ Department of Physiology, College of Medicine, University of Bisha, Bisha, Saudi Arabia; ^2^ Department of Engineering Process, National Higher Polytechnic School of Douala, University of Douala, Douala, Cameroon; ^3^ Department of Pharmacology, College of Medicine, University of Bisha, Bisha, Saudi Arabia; ^4^ Department of Child Health College of Medicine, University of Bisha, Bisha, Saudi Arabia; ^5^ Neoteriks Health Research and Innovation, Avon, IN, United States; ^6^ Department of Basic Medical Sciences, Faculty of Medicine, Majmaah University, Al Majma’ah, Saudi Arabia; ^7^ Department of Pathology College of Medicine University of Bisha, Bisha, Saudi Arabia; ^8^ Department of Internal Medicine, College of Medicine, University of Bisha, Bisha, Saudi Arabia; ^9^ Department of Surgery, College of Medicine, University of Bisha, Bisha, Saudi Arabia; ^10^ Department of Ophthalmology, College of Medicine, King Khalid University, Abha, Saudi Arabia; ^11^ Department of Anatomy, College of Medicine, University of Bisha, Bisha, Saudi Arabia; ^12^ Department of Community and Family Medicine, College of Medicine University of Bisha, Bisha, Saudi Arabia; ^13^ Department of Biochemistry, Faculty of Science, University of Dschang, Dschang, Cameroon

**Keywords:** benzophenone, xanthone, nitric oxide, lipoxygenase, cyclooxygenase, cytokines

## Abstract

**Introduction:** There is a growing interest in studying natural products for the identification of novel lead compounds for drug development for treating inflammatory diseases. Although some studies have focused anti-inflammatory activity of benzophenones and xanthones, exploring additional targets such as enzymes and cytokines, involved in their inflammatory response could provide more comprehensive understanding of the compounds’ anti-inflammatory effects. In this study, four xanthones ananixanthone (**1**), smeathxanthone A (**2**), smeathxanthone B (**3**), and 1,3,5,8-tetrahydroxy-2-(3-methybut-2-enyl)-4-(3,7-dimethyloct-2,6-dienyl) xanthone (**4**); and three benzophenones guttiferone O (**5**), guttiferone M (**6**), and aristophenone A (**7**) from *Garcinia smeathmannii* (Planch. & Triana) Oliv. were investigated for their effect on nitric oxide production, cyclooxygenase, lipoxygenase inhibition, and Th1/Th2 cytokines production in activated RAW 264.7 macrophages.

**Methods:** The Griess reagent method and the ferrous oxidation-xylenol orange assay were used to evaluate the inhibition of NO production and the 15-lipoxygenase activity respectively. Cyclooxygenase activity was assessed using the fluorometric COX activity assay kit and measurement of Th1/Th2 cytokines was performed using a flow cytometer.

**Results:** All the tested compounds exhibited a dose-dependent inhibition of NO production with varying degrees of inhibitory effects on 15-LOX activity. Compound (**6**), displays the best inhibitory effect on COX-1/COX-2 activity. A general trend of the tested compounds on cytokines profiles revealed that compound (**5**) showed a pronounced enhancement of anti-inflammatory cytokines (IL-4 and IL-10).

**Conclusion:** This observation supports future exploration of ananixanthone (**1**), guttiferone O (**5**), and guttiferone (**6**) as potential candidates for the development of anti-inflammatory drugs.

## 1 Introduction

Dysregulated inflammation is a complex biological response orchestrated by the immune system which can lead to a myriad of chronic diseases, including autoimmune disorders, cardiovascular complications, and various cancers (Calhelha et al., 2023). It involves the activation of immune and inflammatory cells such as macrophages (Kolliniati et al., 2022). Macrophages play a crucial role in the initiation and development of multiple inflammatory diseases and could be activated to release inflammatory cytokines and mediators such as nitric oxide (NO), interleukins, interferon-γ (IFN-γ), and tumour necrosis factor-α (TNF-α) (Marrocco and Ortiz, 2022). Additionally, inflammatory enzymes cyclooxygenase (COX) and lipoxygenase (LOX) play pivotal roles in the initiation and progression of inflammation (Cui et al., 2022). Cyclooxygenase, existing in two isoforms (COX-1 and COX-2), catalyzes the conversion of arachidonic acid into prostaglandins, while LOX is responsible for the synthesis of leukotrienes (Smith et al., 2011). Available literature data indicated that pro-inflammatory enzymes and cytokines play an important role in the pathogenesis of inflammation via different pathways (Chen et al., 2018). Hence, the inhibition of these enzymes and other inflammatory mediators is considered an important target for the management of inflammation-related diseases.

The current therapeutic strategy for managing chronic inflammation is largely dominated by synthetic drugs especially non-steroidal anti-inflammatory drugs (NSAIDs). These drugs are often accompanied by limited efficacy and unintended side effects including gastrointestinal complications and immunosuppression (Ziesenitz et al., 2022; Farah et al., 2023). Therefore, there is a need to explore safer and more effective alternatives to overcome painful and other inflammatory conditions. Hence, there has been an increasing interest in the exploration of naturally occurring compounds as potential anti-inflammatory agents (Bakar et al., 2018). Furthermore, a particular focus has emerged on the study of naturally occurring benzophenones and xanthones which are compounds abundant in various plants, and renowned for their diverse biological activities (Xue et al., 2020). Due to their interesting scaffolds and great pharmacological importance, some benzophenone and xanthone compounds have been investigated as potential anti-inflammatory drug candidates (Huang et al., 2020). The ability of several benzophenones and xanthones derivates compounds to alleviate inflammation has been demonstrated so far, with several studies pointing out inhibition of COX enzymes and inflammatory mediators as the main anti-inflammatory mechanisms. 1,3,5,6-tetrahydroxyxanthone (3) and guttiferone E (7) were reported as potential inhibitors of NO production and 15-LOX activity (Dzoyem et al., 2015). In the study of Folquitto et al. (2022), some benzophenones analogs including 2′-hydroxy-4′-benzoylphenyl-β-D-glucopyranoside, 4-hydroxy-4′-methoxybenzophenone and 4′-(4″-methoxybenzoyl) phenyl-β-D-glucopyranoside showed interestig values of Glide Score in COX-2 docking evaluation and selectively inhibited COX-2 and COX-1 in an vitro enzymatic assay (Folquitto et al., 2022). Malik et al. (2022) showed the ability of some benzophenone derivatives to inhibit pro-inflammatory mediators including IFN-γ, TNF-α, IL-1β, IL-6, GM-CSF, IL-2and nitric oxide (NO) (Dzoyem et al., 2015; Folquitto et al., 2022; Malik et al., 2022; Saikia et al., 2023). Another study reported the potential of hydroxyxanthone derivatives to inibit COX-2 enzyme (Dzoyem et al., 2015; Folquitto et al., 2022; Malik et al., 2022; Saikia et al., 2023). Despite these previous work, the full anti-inflammatory potential of the great diversity of benzophenones and xanthones has not yet been fully investigated, exploring additional targets such as enzymes and cytokines, involved in their inflammatory response could provide more comprehensive understanding of the compounds’ anti-inflammatory effects. Therefore, new anti-inflammatory agents having multitarget mechanisms with improved safety profiles that prevent the release of prostaglandins, leukotrienes, and Th1/Th2 cytokines are needed. Therefore, this work was undertaken to investigate seven naturally occurring benzophenones and xanthones derivatives isolated from *Garcinia smeathmannii* (Planch. & Triana) Oliv. as potent anti-inflammatory agents with a different probable mechanism of action. Although several studies have demonstrated the ability of some benzophenones and xanthones to modulate various targets involved in the inflammatory response, detailed evaluation of ananixanthone, smeathxanthones A and B, 1,3,5,8-tetrahydroxy-2-(3-methybut-2-enyl)-4-(3,7-dimethyloct-2,6-dienyl) xanthone, guttiferone O and M, and aristophenone in modulating key mediators of inflammation such as LOX, COX and cytokines has not been carried out. In order to explore the role of the above mentioned benzophenones and xanthones in managing inflammation associated with various diseases, this study was designed to investigate *in vitro* effect on nitric oxide production, COX/15-LOX inhibition and Th1/Th2 cytokines production in RAW264.7 activated macrophages.

## 2 Materials and methods

### 2.1 Natural products

#### 2.1.1 Plant material

The stem bark of *Garcinia. smeathmannii* (Planch. & Triana) Oliv. was collected from Cheffou-Baham (5° 20′9.33″N, 10° 23′34.84″E), western province, Cameroon in April 2010 and was identified by Victor Nana of the Cameroon National Herbarium (CNH), Yaoundé, where a voucher specimen (35169/HNC) has been deposited.

#### 2.1.2 General experimental procedures

Melting points were determined on a Büchi-540 melting point apparatus. IR spectra were determined on Nicolet 380 Fourier Transform IR spectrometer. UV spectra were determined on a Spectronic Unicam spectrophotometer. The ^1^H, ^13^C, and DEPT NMR spectra, as well as two-dimensional experiments (COSY, NOESY, HSQC, HMBC using pulsed field gradients), were recorded on a Bruker DRX 500 FT-NMR spectrometer, operating at 500 MHz (^1^H) and 125 MHz (^13^C) and *Avance* 600 FT-NMR spectrometer, operating at 600 MHz (^1^H) and 150 MHz (^13^C) in CDCl_3_, C_3_D_6_O or DMSO-d_6_ with TMS as internal standard.

EI Mass spectra and accurate mass spectra were recorded with a LC linear ion trap instrument (Esquire 3000) using electrospray ionization in the negative or positive mode and Sector field mass spectrometer (Autospec X). Vacuum Liquid Chromatography (VLC) was carried out using Merck Silica Gel 60 GF254 (230–400 mesh), column chromatography using Silica Gel 60 (230–400 mesh, 70–230 mesh), TLC analysis was performed on Silica Gel plates (Merck kieselgel 60 GF254, 0.25 mm, 20 × 20 cm) with different mixtures of petrol ether, cyclohexane, dichloromethane, ethyl acetate, and acetone and methanol as eluents; spots were visualized under UV lamps (254 nm) and (365 nm) or by MeOH–H_2_SO_4_ reagent. Solvent evaporation was done using Rota vapor (laborota 4000; Heidolph).

#### 2.1.3 Isolation and identification of compounds

The air-dried, powdered stem bark of *G. smeathmannii* (2.5 kg) was extracted at room temperature for 3 days using distilled methanol (12 L). The crude methanol extract (207 g) obtained was partitioned with petroleum ether (88 g; 2.5 L), dichloromethane (20.4 g; 1.5 L), and ethyl acetate (32 g; 2 L).

The petroleum ether fraction (80 g) was subjected to flash column chromatography using silica gel (230–400 mesh; 800 g) eluted with pure petroleum ether, petroleum ether-EtOAc (9: 1), petroleum ether-EtOAc (7.5: 2.5), petroleum ether-EtOAc (1: 1), EtOAc and EtOAc-MeOH (7.5: 2.5) to give five main fractions labeled A (27 g), B (22 g), C (14 g), D (17 g), and E (6.4 g), respectively.

Fraction A (25 g) was then subjected to column chromatography (5 × 100 cm) on silica gel (600 g, 230–400 mesh) and eluted by a petroleum ether-EtOAc mixture of increasing polarity (20: 1–3: 1). A total of 90 fractions of ca. 300 mL each were collected, concentrated and combined based on TLC to give five subfractions indexed A1 to A4. The subfraction A3 (1.9 g) was further subjected to column chromatography (4 × 30 cm) on silica gel (25 g, 70–230 mesh) and eluted with petroleum ether-EtOAc (18: 2) to yield compound **4** (8 mg, ≥98% purity). The subfraction A4 (19.2 g), after column chromatography (5 × 50 cm) on silica gel (350 g, 70–230 mesh), yielded a mixture of polyprenylated benzophenones. Fractions were then mixed again and submitted to LH-20 Sephadex column chromatography (1.5 × 150 cm), eluted with a mixture of DCM-MeOH (1:1) to afford compound **5** (13.5 mg, ≥95% purity) and **6** (24 mg, ≥98% purity).

Fraction B (20 g) was also subjected to column chromatography (5 × 100 cm) on silica gel (600 g, 230–400 mesh) and eluted with a petroleum ether-EtOAc mixture of increasing polarity (20: 1–3: 1). Fifty fractions of ca. 300 mL each were collected and regrouped into three subfractions, B1 to B3, based on their TLC profile. Subfraction B1 (1.8 g) was subjected to column chromatography (4 × 30 cm) on silica gel (20.0 g, 70–230 mesh) and eluted with a mixture of petroleum ether-EtOAc (17: 3) to give compound **3** (5.0 mg, ≥99% purity). Subfraction B2 (8.0 g) was subjected to column chromatography (4 × 30 cm) on silica gel (20 g, 70–230 mesh) and eluted with a mixture of petroleum ether-EtOAc (13: 7) to yield compounds **1** (25.0 mg, ≥97% purity). Subfraction B3 (2.6 g) was also subjected to column chromatography (4 × 30 cm) on silica gel (30 g, 70–230 mesh) and eluted with a mixture of petroleum ether- EtOAc (12: 8) to afford compounds **7** (15.0 mg, ≥98% purity) and **2** (22.5 mg, ≥98% purity).

*Ananixanthone (1)*: pale colorless amorphous, mp 107-171 (MeOH); UV (MeOH) λ_max_ (logε) 253.3 (4.19), 269.6 (4.19), 332.8 (3.74); UV (MeOH + NaOH) λ_max_ (logε) 269.6 (4.23), 285 (4.13) sh, 348 (3.62); UV (MeOH + NaOAc) λ_max_ (logε) 269.6 (4.27), 285 (4.15) sh, 338.3 (3.62); UV (MeOH + AlCl_3_) λ_max_ (logε) unchanged. IR (KBr) ν_max_: 3377, 2919, 2852, 1648 cm^−1^ (Bayma et al., 1998).

*Smeathxanthone A (2):* Yellow crystals, mp. 216°C–218°C; UV (EtOH) λ_max_ (logε): 408 (0.32), 338 (0.21), 337 (0.78), 297 (2.63), 223 (1.27), 205 (5.00); IR (KBr) ν_max_: 3315, 2891, 2350, 2200, 1962, 1869, 1579, 1440, 1290, 1193, 1084, 936, 822, 784 cm^−1^ (Komguem et al., 2005).

*Smeathxanthone B (3):* Yellow powder; mp. 187°C–189°C; ${\left[\alpha \right]}_{D}^{22}$ + 30.3 (c, 0.02 MeOH); UV (EtOH) λ_max_ (logε): 408 (0.32), 338 (0.21), 337 (0.78), 297 (2.63), 223 (1.27), 205 (5.00); IR (KBr) ν_max_: 3727, 3414, 2965, 2359, 2262, 2062, 1987, 1636, 1586, 1346, 1052, 1000, 935, 862, 774 cm^−1^ (Komguem et al., 2005).

*1,3,5,8-tetrahydroxy-2-(3-methybut-2-enyl)-4-(3,7-dimethyloct-2,6-dienyl) xanthone (4):* yellow powder, mp 172°C–173°C; UV (MeOH) λ_max_ (logε): 283 (4.6), 325 (4.2),350 (4.3) 400 (3.8) nm; IR (solid) ν_max_: 2919, 2851, 1627, 1617, 1580, 1485, 1463, 1314, 1217, 1176, 1099, 1007, 967, 830, 809, 719, 704, 618, 589 cm^−1^ (Fouotsa et al., 2015).

*Guttiferone O (5):* Yellow powder; mp: 103°C; ${\left[\alpha \right]}_{D}^{20}:$ +45 (c 0.17,CH_3_COCH_3_); UV(MeOH): λ(logε) = 281 (3.03), 229 (3.04), 297 (2.50) nm; IR (KBr): ν_max_ = 3360, 1725, 1642, 1627 cm^−1^ (Lannang et al., 2010).

*Guttiferone M (6):* yellow oil; ${\left[\alpha \right]}_{D}^{24}:$ −29.8 (MeOH; c 0.15); UV (MeOH) λ_max_ (logε) 230 (sh), 280 (3.80), 355 (sh) nm; IR (KBr) ν_max_: 3425, 2936, 1715, 1641, 1225, 1060 cm^−1^ (Masullo et al., 2008).

*Aristophenone A (7):* yellow cubes (CHCl3); mp. 82°C; ${\left[\alpha \right]}_{D}^{25}$ +58° (c 0.1, CHCl_3_); UV (EtOH) λ _max_ (log𝞮) 280 (4.21) and 228 (4.34) nm; IR (KBr) ν _max_ 3350, 1730, 1715, 1644, 1220, 1151 cm^−1^ (Cuesta-Rubio et al., 2001).

### 2.2 Chemicals

Lipopolysaccharide (LPS) from *Escherichia coli* 0111:B4 was obtained from Sigma-Aldrich, Darmstadt, Germany. Penicillin/streptomycin/fungizone (PSF), Dulbecco’s modified Eagle’s medium (DMEM), and fetal calf serum (FCS) were purchased from Highveld Biological Products (South Africa). Whitehead Scientific (South Africa) provided trypsin and phosphate-buffered saline (PBS). 3-(4, 5-dimethylthiazol-2-yl)-2, 5-diphenyl-tetrazolium bromide (MTT) and quercetin were provided by Sigma-Aldrich St. Louis, MO, United States. Linoleic acid was purchased from Merck (Darmstadt), xylenol orange from Searle (England), and sodium carbonate from Holpro Analytic (South Africa). Tris (hydroxymethyl) aminomethane was purchased from Sigma, (Switzerland) while 15-lipoxygenase from *Glycine max,* ferrous sulfate, indomethacin, and sodium nitrite were obtained from Sigma (Germany).

### 2.3 Nitric oxide inhibitory activity in LPS-activated RAW 264.7 macrophages

#### 2.3.1 Cell culture and maintenance

Murine macrophage RAW 264.7 cell line obtained from the American Type Culture Collection (Rockville, MD, United States) was maintained in DMEM supplemented with 10% fetal calf serum (FCS) and 1% penicillin/streptomycin/fungizone (PSF) under standard cell culture conditions at 37°C and 5% CO_2_ in a humidified environment.

#### 2.3.2 Cytotoxicity assay

The cytotoxic effect of compounds on RAW 264.7 cells was assessed using the 3-(4, 5-dimethylthiazol-2-yl)-2, 5-diphenyl tetrazolium bromide (MTT) assay as established by Mosmann (Mosmann, 1983) with slight modifications. Briefly, cells were seeded at a density of 1 × 10^5^ cells/mL (100 µL) in 96-well microtitre plates and incubated at 37°C and 5% CO_2_ in a humidified environment. After 24 h incubation, 100 µL of compounds (at concentration ranged from to 250 μM–1.95 µM) were added to the wells containing cells and further incubated for 48 h in a CO_2_ incubator. A suitable control (untreated cells) was included and doxorubicin was used as a positive reference. Thereafter, the medium in each well was aspirated from the cells, which were then washed with PBS, and finally fresh medium (200 µL) was added to each well. Then, 30 µL of MTT (5 mg/mL in PBS) was added to each well and the plates were incubated at 37°C for 4 h. The medium was aspirated from the wells and DMSO was added to solubilize the formed formazan crystals. The absorbance was read at 570 nm (SpectraMax 190, Molecular devices). The percentage of cell inhibition was calculated with reference to the control (untreated cells taken as 100% viability). Then, the IC_50_ values of plant compounds showing more than 50% cell growth inhibition were calculated by plotting the percentage inhibition against the concentration.

#### 2.3.3 Inhibition of nitric oxide (NO) production in LPS-activated RAW 264.7 macrophages

The RAW 264.7 macrophage cells were seeded in 96 well-microtitre plates (2 × 10^5^ cells/mL). They were activated by incubation in a medium containing 1 μg/mL LPS alone (control) or lipopolysaccharide, and compounds at concentration of 3.12 µM, 6.25 µM, 12.5 µM and 25 µM dissolved in DMSO. Quercetin served as a positive control NO inhibitor for the reduction of NO production. After 24h, the amount of nitric oxide released from macrophages was determined using the Griess reagent as previously described (Dzoyem and Eloff, 2015).

### 2.4 Soybean 15-LOX inhibition assay

The assay was performed according to a previously described procedure (Pinto et al., 2007) with slight modifications. The assay is based on measuring the formation of the complex Fe3^+^/xylenol orange in a spectrophotometer at 560 nm. 15-LOX from *G. max* was incubated with compounds or standard inhibitors at 25°C for 5 min. The concentration range of the samples were from 250 μM to 1.95 µM. Then linoleic acid (final concentration, 140 µM) in Tris-HCl buffer (50 mM, pH 7.4) was added and the mixture was incubated at 25°C for 20 min in the dark. The assay was terminated by the addition of 100 µL of FOX reagent consisting of sulfuric acid (30 mM), xylenol orange (100 µM), iron (II) sulfate (100 µM) in methanol/water (9:1). For the control, only LOX solution and buffer were pipetted into the wells. Blanks (background) contained the enzyme LOX during incubation, but the substrate (linoleic acid) was added after the FOX reagent. Quercetin was used as a reference compound for the inhibition of 15-LOX activity (Sadik et al., 2003). The LOX inhibitory activity was evaluated by calculating the percentage of the inhibition of hydroperoxide production from the changes in absorbance values at 560 nm after 30 min at 25°C. % inhibition = [(A_control_ – A_blank_) – (A_sample_ – A_blank_)/(A_control_ – A_blank_)] x100. Where A_control_ is the absorbance of control well, A_blank_ is the absorbance of blank well and A_sample_ is the absorbance of sample well.

### 2.5 Evaluation of COX-1 and COX-2 activity

The fluorometric cyclooxygenase activity assay kit (Biovision) was used to evaluate the activity of COX-1 and COX-2 enzymes. Raw 264.7 cells were seeded at 2 × 10^5^ cells/mL in a 48-well microplate, and allowed to adhere for 24 h, then treated with LPS 0.1 μg/mL and compounds **(1)**, **(5)** and **(6)** at 20 µM against Raw 264.7 cells. After 24 h the cyclooxygenase enzyme (COX-1 and COX-2) activity assay was performed. Cells were detached with TNE buffer (Tris 40 mM, NaCl 150 mM, EDTA 1 mM) and washed with PBS (1x), then re-suspended in 1 mL PBS (1x), transferred into 1.5 mL tube, and centrifuged at 500 x g for 3 min. The pellet was then re-suspended in 0.5 mL of lysis buffer with a protease inhibitor cocktail, vortexed, and incubated at 4°C for 5 min. The cell lysate was centrifuged at 12000 x g for 3 min and the supernatant was collected for COX activity assay. The COX activity was assessed using the fluorometric cyclooxygenase activity assay kit (Biovision) following the manufacturer’s instructions. The assay includes COX-1 and COX-2 specific inhibitors to differentiate the activity of COX-1 and COX-2 as well as other peroxidases. Indomethacin (10 µM) was used as a standard drug and appropriate controls were also included.

### 2.6 Measurement of Th1/Th2 cytokines

Raw 264.7 cells were seeded at 2 × 10^5^ cells/mL in a 48-well microplate and allowed to adhere for 24 h, then treated with LPS 0.1 μg/mL and with compounds **(1)**, **(5)** and **(6)** at 20 µM. Untreated cells and indomethacin (10 µM) were used as controls. After 24 h of incubation, the supernatant was collected and the amount of pro-inflammatory Th1 cytokines (IFN-γ, TNF-α, and IL-2) and anti-inflammatory Th2 cytokines (IL-4, IL-6, and IL-10) released measured. The experiment was performed according to the manufacturer’s instructions using BD™ Cytometric Bead Array (CBA) Human Th1/Th2 Cytokine Kit (BD-Biosciences), and data were acquired on a BD LSR Fortessa™ cell analyzer flow cytometer.

### 2.7 Statistical analysis

The data are presented as the mean ± standard deviation (SD) of three independent experiments or triplicate (*n* = 3). Differences between the means of each group were assessed by a one-way analysis of variance followed by Sidak’s multiple comparisons test using GraphPad Prism 9.

## 3 Results

### 3.1 Isolation and structural elucidation of compounds

**FIGURE 1:**
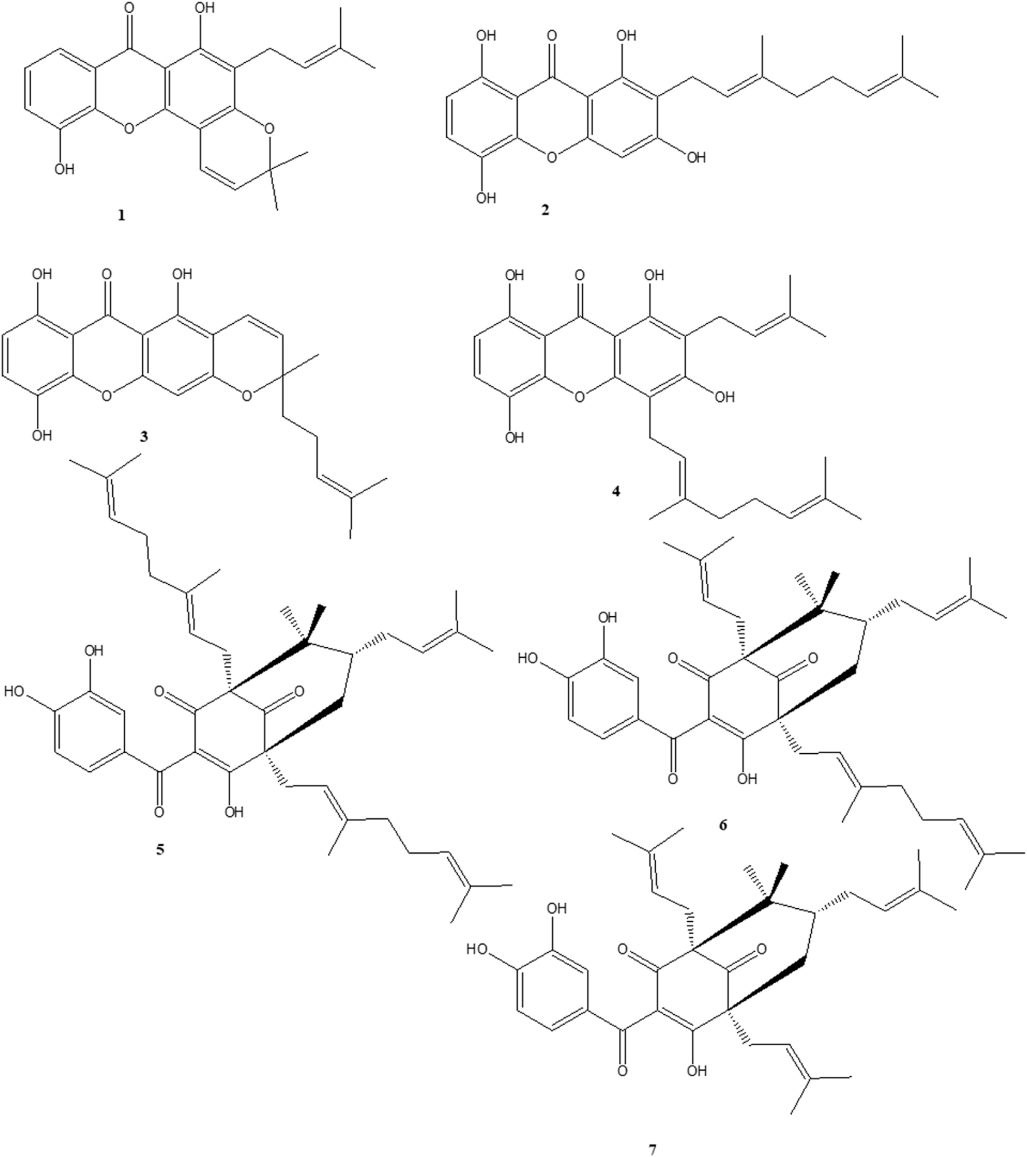
Chemical structures of naturally occurring benzophenones and xanthones. ananixanthone **(1)**, smeathxanthone A **(2)**, smeathxanthone B **(3)**, 1,3,5,8-tetrahydroxy-2-(3-methybut-2-enyl)-4-(3,7-dimethyloct-2,6-dienyl) xanthone **(4)**, guttiferone O **(5)**, guttiferone M **(6)**, and aristophenone A **(7)**.

Extensive column chromatography on methanol (MeOH) extract of the stem bark of *Garcinia Smeathmannii* (Planch. & Triana) Oliv. using silica gel and Sephadex LH-20 led to the isolation of seven known compounds (**1–7**). Their structures were established through spectroscopic (1D, 2D NMR) data, spectrometric analysis (MS), and by comparison with literature data. Isolated compounds were identified by comparison of their spectroscopic data with literature values. They are Ananixanthone **(1)**, Smeathxanthone A **(2)**, Smeathxanthone B **(3)**, 1,3,5,8-tetrahydroxy-2-(3-methybut-2-enyl)-4-(3,7-dimethyloct-2,6-dienyl) xanthone **(4)**, Guttiferone O **(5)**, Guttiferone M **(6)**, Aristophenone A **(7)** (Figure 1).

### 3.2 Cytotoxicity of compounds against RAW 264.7 macrophages

**FIGURE 2:**
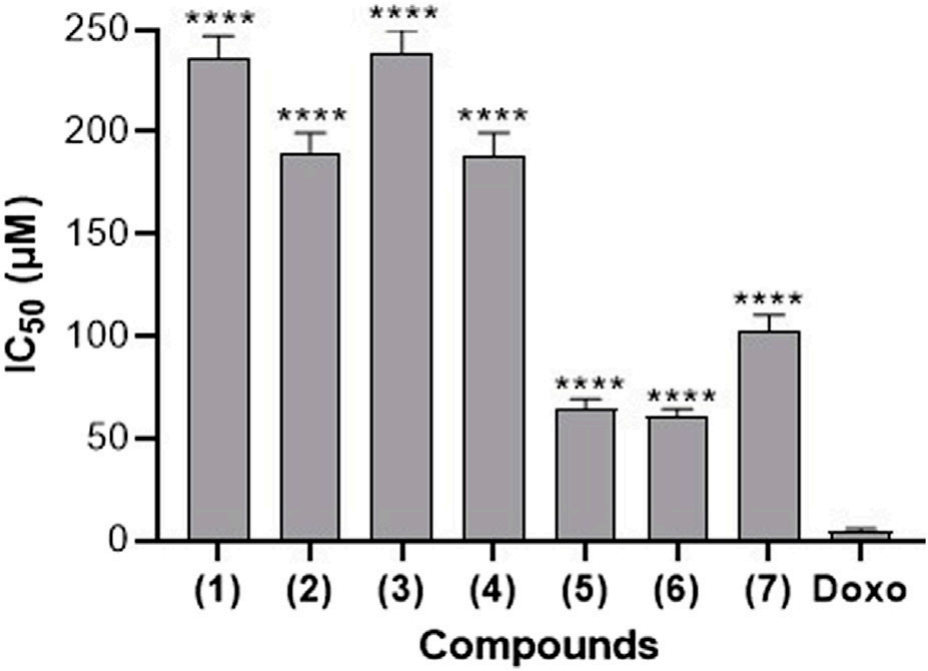
Cytotoxic effect of seven naturally occurring benzophenones and xanthones from *Garcinia smeathmannii* on RAW264.7 cells. Data are the mean from three independent experiments. Sidak’s multiple comparisons test using one-way ANOVA was performed: ∗∗∗∗*p* < 0.0001 for compounds *versus* reference drug doxorubicin (Doxo).

In order to assess the safety of the investigated compounds towards the RAW 264.7 cells and to select the sub-inhibitory concentration for further experiments, a cytotoxicity assay was conducted and the results are presented in Figure 2. It appears from this Figure 2 that, compounds **(1)**, **(3)**, and **(7)** exhibit relatively higher IC_50_ values. These values are above 100 μM, suggesting that they might have lesser cytotoxic effects on RAW264.7 cells compared to the other compounds. Compounds **(5)** and **(6)** have the lowest IC_50_ values (64.54 µM and 61.25 µM respectively), indicating greater potency in inhibiting cell growth or inducing cell death in RAW264.7 cells. Doxorubicin, a known chemotherapeutic drug with cytotoxic properties, serves as a reference. It exhibits an IC_50_ value of 4.63 µM, which is lower than all the compounds tested. Based on these data, sub-inhibitory concentration of 25 µM was chosen for NO assay.

### 3.3 Nitric oxide (NO) production inhibition and cell viability

**FIGURE 3:**
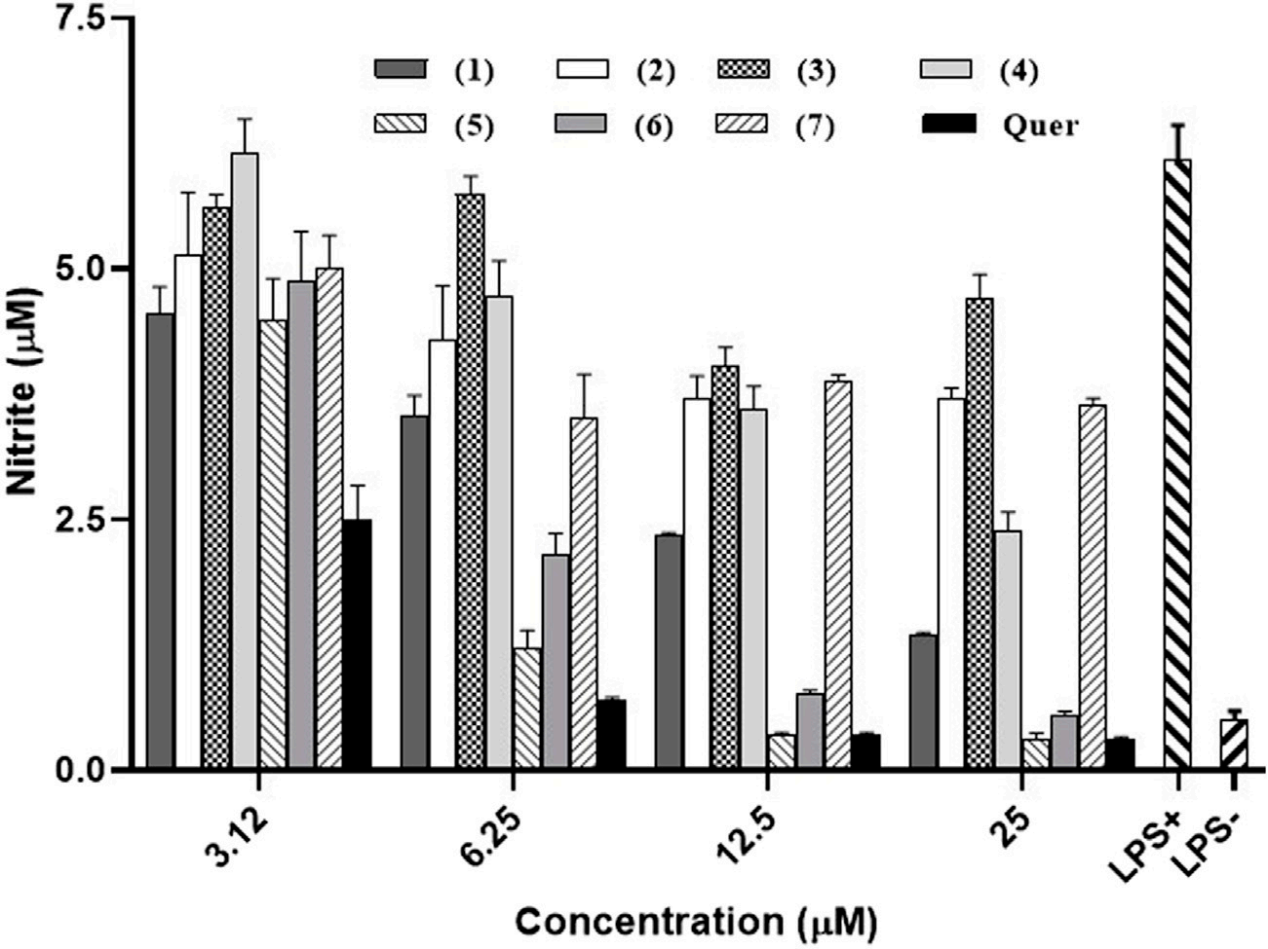
Inhibitory activities of seven naturally occurring benzophenones and xanthones from *Garcinia smeathmannii* on NO production in LPS-stimulated in RAW 264.7 macrophages. Quer: quercetin.

The ability of compounds obtained from *G. smeathmannii*, to inhibit NO production in the RAW 264.7 cell line after stimulation with lipopolysaccharide (LPS) was evaluated and results are shown in Figure 3. All the tested compounds exhibited a dose-dependent inhibition of NO production. At the highest concentration (25 µM), guttiferone O **(5)**, and guttiferone M **(6)** exhibit the most potent inhibitory activities reducing the amount of NO produced from 6.08 µM in untreated and stimulated control to 0.3 µM and 0.54 µM respectively.

### 3.4 Inhibition of 15-lipoxygenase activity

**FIGURE 4:**
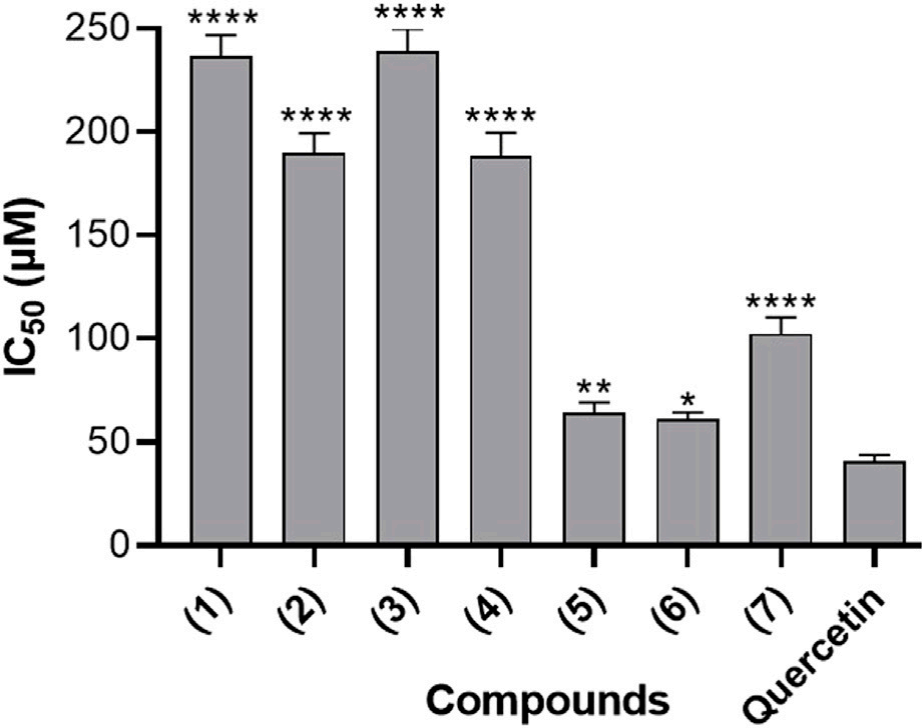
15-Lipoxygenase inhibitory activity of seven naturally occurring benzophenones and xanthones from *Garcinia smeathmannii*. Data are the mean from three independent experiments. Sidak’s multiple comparisons test using one-way ANOVA was performed: ∗∗*p* < 0.01, ∗∗∗*p* < 0.001, and ∗∗∗∗*p* < 0.0001 for compounds *versus* reference compound quercetin (Quer).

Figure 4 presents the inhibitory effects of the seven naturally occurring benzophenones and xanthones on 15-Lipoxygenase (15-LOX) activity, expressed in terms of IC_50_ values. These results suggested that the evaluated compounds possess varying degrees of inhibitory effects on 15-LOX activity. The IC_50_ values obtained ranged between 45.01 μM and 160.73 μM. Ananixanthone (**1**), Guttiferone O (**5**) and Guttiferone M (**6**) demonstrated IC_50_ values of 63.91µM, 45.01 µM and 52.97 µM, respectively, which appears to be the most effective among the seven tested compounds, as compared to quercetin, a well-studied flavonoid, used as a positive control which showed an IC_50_ value of 64.35 µM.

### 3.5 Inhibition of cyclooxygenase enzyme activity

**FIGURE 5:**
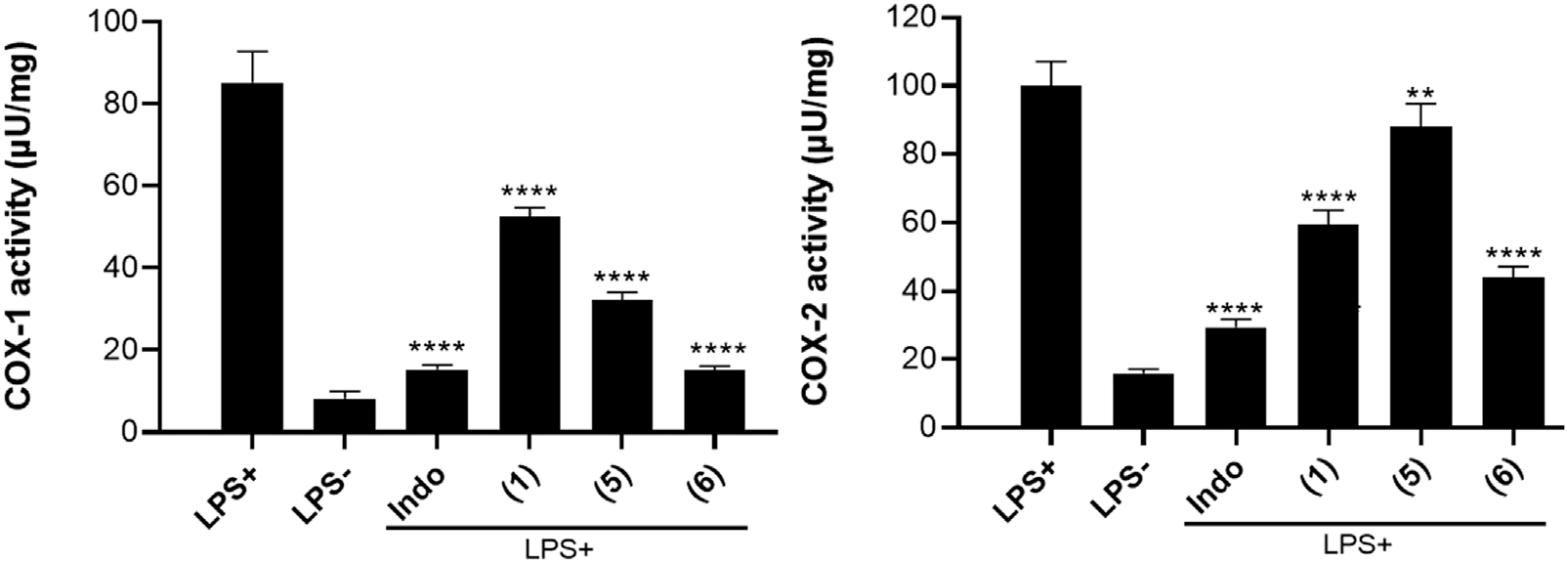
Results of COX-1 and COX-2 activity in LPS-stimulated MH-S cell lysate treated with compounds **(1)**, **(5)** and **(6)** at 20 µM. The positive control indomethacin (Indo) was tested at 10 μM. One unit (U) of COX activity is the amount of enzyme that generates 1.0 μmol of resorufin per minute at pH 8.0°C and 25°C. Data are the mean of three independent experiments. Sidak’s multiple comparisons test using one-way ANOVA was performed: ∗∗*p* < 0.01, ∗∗∗*p* < 0.001, and ∗∗∗∗*p* < 0.0001 for compounds *versus* stimulated and untreated control.

The results of the inhibitory effect of three selected naturally occurring benzophenones and xanthone on COX-1 & COX-2 enzyme activity are provided in Figure 5. A perusal of this figure reveals that all three compounds exhibited some levels of inhibition on COX-1/COX-2 activity. Compared to the untreated controls, all the compounds significantly inhibit the activity of COX-1 (*p* < 0.0001) reducing the activity of the enzyme from 85.19 µU/mg to 52.55 µU/mg, 32.28 µU/mg, and 5.13 µU/mg for compound **(1)**, **(5)** and **(6)** respectively. Compound **(6),** guttiferone M displays the best inhibitory effect on COX-1 with a value (15.13 µU/mg). Likewise, all the compounds significantly inhibit the activity of COX-2 (*p* < 0.0001 for compounds **(1)** and **(6)**, *p* < 0.01 for compound **(5)**). The activity of COX-2 was reduced from 99.85µU/mg in untreated control to 59.33 µU/mg, 88.10 µU/mg, and 43.84 µU/mg for compounds **(1)**, **(5)** and **(6)** respectively.

### 3.6 Modulation of Th1/Th2 cytokines

**FIGURE 6:**
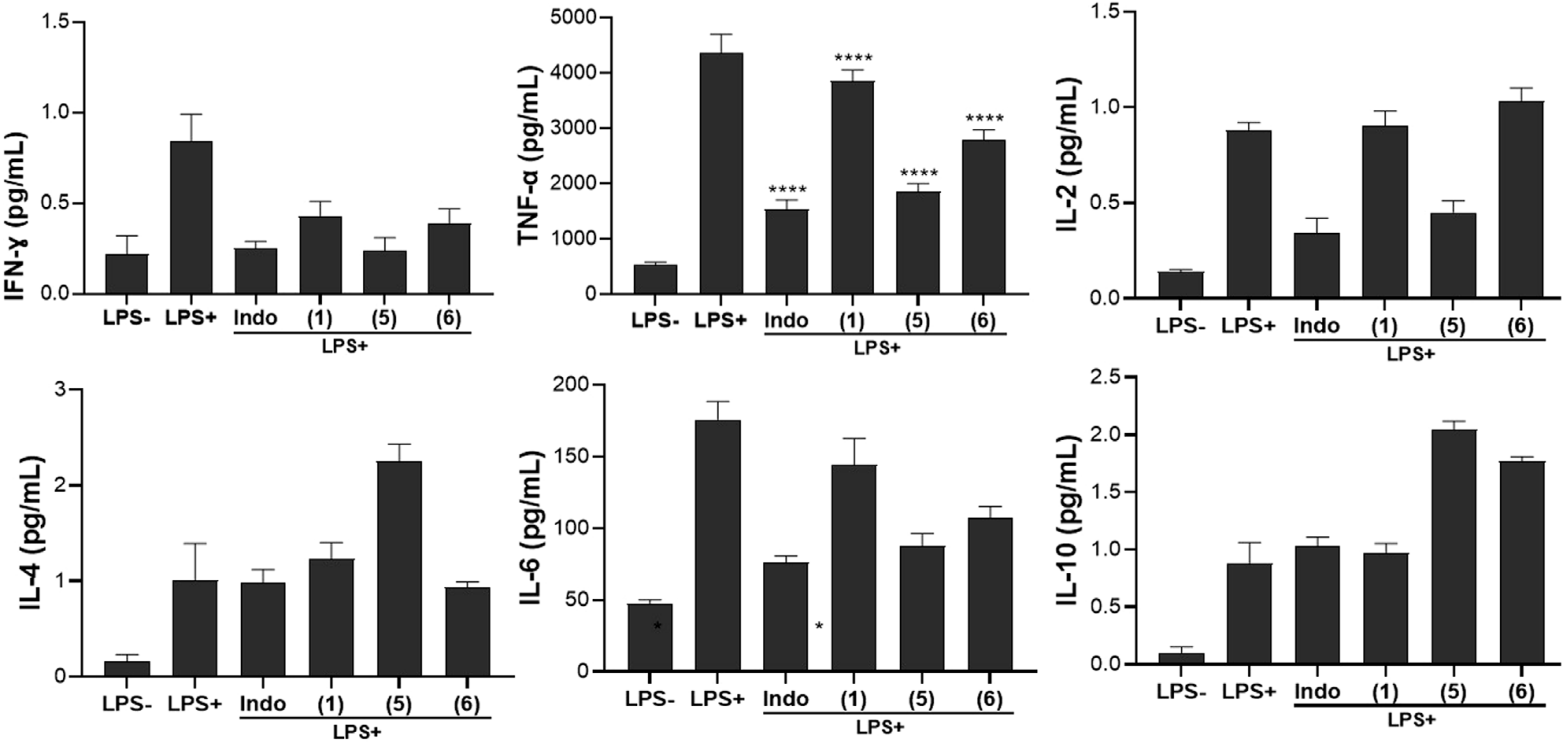
Effect of compounds **(1)**, **(5)**, and **(6)** on inflammatory cytokines production in LPS-stimulated Raw 264.7 macrophage cells. Cells were treated for 24 h with compounds (1), (5) and (6) at 20µM, then cytokines secretion levels were measured. Indomethacin (Indo) was tested at 10 µM. Values are the mean of experiments done in triplicate (*n* = 3) ± standard deviation. Statistical analysis was performed with Sidak’s multiple comparisons test using one-way ANOVA. ∗∗∗∗*p <* 0.0001 between the sample and stimulated and untreated control.

In this work, the effects of compounds **(1)**, **(5),** and **(6)** on the production of human Th1/Th2 cytokines were evaluated. The cytokines measured include pro-inflammatory Th1 cytokines (IFN-γ, TNF-α, IL-2, IL-6) and anti-inflammatory Th1 cytokines (IL-4, IL-10). Results presented in Figure 6 indicated that the compounds tested exhibit diverse effects on the production of the pro- and anti-inflammatory cytokines.

The stimulated and untreated control groups had different levels of pro-inflammatory cytokines viz, 0.84 pg/mL for IFN-γ, 4356.86 pg/mL for TNF-α, and 0.88 pg/mL for IL-2; while the levels of anti-inflammatory cytokines were 1.01 pg/mL, 175.96 pg/mL, and 0.88 pg/mL for IL-4, IL-6, and IL-10, respectively. Upon treatment, compound **(1)** indicated a moderate decrease in IFN-γ and IL-2 production (0.43 pg/mL and 0.90 pg/mL respectively) but had a minimal effect on TNF-α (3854.61 pg/mL) compared to the stimulated and untreated control. This demonstrated a potential anti-inflammatory effect, though not as pronounced as seen with indomethacin.

## 4 Discussion

Nitric oxide (NO) plays a pivotal role in the physiological process of inflammation and has been involved in endotoxin-induced tissue injury due to its overproduction (Goncharov, 2020). Therefore, the inhibition of NO production has been considered a target for anti-inflammatory therapeutics (Wong and Lerner, 2015). Many plants of the *Garcinia* genus containing benzophenones and xanthones as bioactive constituents have been traditionally used as anti-inflammatory agents (Espirito Santo et al., 2020). Among the xanthone derivatives tested, ananixanthone **(1)** shows a remarkable inhibitory effect at the highest concentration (25 µM), suggesting its potential anti-inflammatory activity. Previous studies have highlighted the ability of these compounds to modulate inflammatory pathways, including the inhibition of NO production (Dzoyem et al., 2015). A benzophenone derivative clusiacyclol A was shown to have a significant NO inhibitory activity in RAW 264.7 macrophage cells with over 85% inhibition without obvious cytotoxicity at a final concentration of 100 μM (Sukandar et al., 2023). Similarly, xanthones derivatives garcinoxanthones B and C significantly inhibited NO production with IC_50_ values of 11.3 μM and 18.0 μM respectively (Liu et al., 2016). In this study, the observed inhibitory effects suggest that compounds **(1)**, **(5),** and **(6)**, may interfere with the inflammatory cascade by suppressing NO production.

Lipoxygenase (LOX) and cyclooxygenase (COX) are the enzymes responsible for the conversion of arachidonic acid to eicosanoids which are essential to initiate the immunological response during inflammation (Mukhopadhyay et al., 2023). Targeting the dual inhibition of COX/LOX is a promising strategy to develop novel anti-inflammatory agents (Cheng et al., 2022; Rudrapal et al., 2023). In our continued search for novel, more efficacious, and safer anti-inflammatory agents having dual mechanisms that could prevent the release of both prostaglandins and leukotrienes, benzophenones and xanthones compounds herein investigated. The compounds were screened for their ability to inhibit the activity of 15-LOX, then the most potent were selected and tested for their effect on the inhibition of COX-1 and COX-2 activities. Although not exhaustive, the literature on benzophenones and xanthones compounds supports the idea that some of them exhibit anti-inflammatory properties through the inhibition of LOX enzymes (Yamashita et al., 1990; Santos et al., 2017; Lončarić et al., 2021). The observed IC_50_ values provide insights into the concentration at which these compounds exert their inhibitory effects, allowing for a better understanding of their potential therapeutic relevance. From these results, ananixanthone **(1)** guttiferone O **(5)** and guttiferone M **(6)** which appear as the most effective in inhibiting the activity of 15-LOX were selected emphasized the need for further investigation for their effect on the activity of COX enzymes. This finding is noteworthy as COX-2 is often upregulated in response to inflammatory stimuli, and its inhibition is a key target for anti-inflammatory drugs (Gandhi et al., 2017). Once more, compound **(6)** exhibited the most potent inhibitory effect on COX-2 suggesting its multitarget potential. Although no previous report focusing on the effect of compounds **(1)**, **(5)** and **(6)** on COX enzyme activity is documented, the observed inhibitory activity on both COX-1 and COX-2 is consistent with the broader literature on benzophenones and xanthones derivatives. The benzophenones and xanthones group of compounds has been recognized for their anti-inflammatory potential, and the modulation of COX as the plausible mechanism underlying their effects (Miladiyah et al., 2017; Qiao et al., 2021; Dzoyem et al., 2022). The observation that compound (6) had comparable result with the indomethacin positive controls was encouraging, as it suggests the potential for developing novel anti-inflammatory agents. Moreover, some well-known NSAIDs such as ibuprofen and aspirin (Acetylsalicylic Acid) are non-selective inhibitors of cyclooxygenase (COX) by reducing the production of prostaglandins, which are mediators of inflammation and pain.

Cytokines are endogenous mediators that play an important role in the pathophysiology of the systemic inflammatory response (Ishii and Yoshida, 2010). The production of both pro- and anti-inflammatory cytokines is strictly controlled by complex feedback mechanisms (Kishen and Muralidharan, 2020). Pro-inflammatory cytokines are responsible for initiating an effective inflammatory process against pathogens, whereas their overproduction has been associated with harmful effects on the body (Cole et al., 2018). In contrast, anti-inflammatory cytokines are involved in down-regulating the exacerbated inflammatory response and maintaining homeostasis (Ishii and Yoshida, 2010; Muzamil et al., 2021). Cytokines are mainly produced by macrophages and lymphocytes. In this study we used RAW 264.77 cells as model for cytokines production after stimulation with lipopolysaccharide (LPS) from *E. coli* 0111:B4. The result obtained with compound **(1)** corroborated with the increase in the levels of the anti-inflammatory cytokine IL-4 (1.23 pg/mL). However, its effect was minimal towards IL-10 (0.971 pg/mL) and there was a reduction in IL-6 (144.25 pg/mL), indicating instead its potential modulation of pro-inflammatory responses. To the best of our knowledge, this is the first study reporting the effect of ananixanthone on cytokine production. However, several xanthone derivatives have been shown to regulate various inflammatory activities and signaling pathways in immune cells, especially macrophages (Ng and Chua, 2019; Feng et al., 2020). Another xanthone derivative, α-mangostin was shown to inhibit the production of interleukin (IL)-6 (Ibrahim et al., 2016). The results of the effect of the two benzophenone compounds tested showed that compound **(5)** significantly reduces the production of IFN-γ, TNF-α, and IL-2 cytokines, similar to the effect of Indomethacin. This suggests a strong anti-inflammatory impact, potentially inhibiting the inflammatory response induced by LPS. Compound **(6)** exhibited a moderate decrease in IFN-γ and TNF-α production but had a limited effect on IL-2 compared to the stimulated and untreated control, indicating a partial anti-inflammatory effect, with a more substantial impact on IFN-γ and TNF-α. A substantial increase in the production of both IL-4 (2.25 pg/mL) and IL-10 (2.05 pg/mL) upon treatment with compound **(5)** was observed, indicating a robust enhancement of anti-inflammatory cytokines. Compound **(6)** had a moderate effect on IL-4 (0.94 pg/mL) and IL-10 (1.77 pg/mL). A general trend of the tested compounds on cytokines profiles revealed that compound **(5)** showed a pronounced enhancement of anti-inflammatory cytokines (IL-4 and IL-10), suggesting a potent anti-inflammatory effect while compound **(6)** exhibits a moderate effect on anti-inflammatory cytokines. Previous studies reporting the anti-inflammatory effect of benzophenones via modulation of cytokines are very scarce. However, a study by Rachoń et al. showed that benzophenone-2 was able to shift the Th1/Th2 balance toward a Th2 response (lower IFN-γ production and higher IL-10) (Rachoń et al., 2006). Another study showed that a 4-aminobenzophenone derivative displayed potent inhibition against TNF-α and IL-1β in LPS-stimulated human peripheral blood mononuclear cells (Khanum et al., 2004). Another benzophenone derivative otogirinin A was reported to suppress TNF-α generation via blocking the phosphorylation of MAPK/JNK and degradation of IκBα (Huang et al., 2020). Overall, the results in Figure 6 highlight the modulatory effects of the three selected compounds on inflammatory cytokine production. The observed changes in cytokine levels indicate potential anti-inflammatory and modulatory properties, emphasizing the need for further studies to elucidate the underlying mechanisms and validate the therapeutic potential of these compounds in the context of inflammatory disorders.

## 5 Conclusion

In this study, we have shown that compounds **(1)**, **(5)** and **(6)** exerted anti-inflammatory by inhibition of NO, inhibition of the activity LOX, COX-1, and COX-2, as well as by regulating the production of cytokines. These data contributed to the growing body of evidence supporting the anti-inflammatory potential of benzophenones and xanthones derivatives and the use of natural compounds in the development of novel anti-inflammatory therapeutics. Our findings further support the exploration of ananixanthone **(1)**, guttiferone O **(5),** and guttiferone **(6)** as potential candidates for the development of anti-inflammatory drugs. Although this study lacks information on the mechanism underlying the inhibitory effects of psoralen on the synthesis of cyclooxygenase enzymes and the studied of other inflammatory cytokines. Results are promising and further studies, including *in vivo* experiments, exploration of the signaling pathways such as NF-κB and MAPKs, synergistic effects of psoralen with other isolated compounds, are needed to confirm the efficacy and safety of psoralen as an anti-inflammatory agent.

## Data Availability

The original contributions presented in the study are included in the article/Supplementary Material, further inquiries can be directed to the corresponding authors.
